# Liver teratoma in a 14-year-old female: a case report

**DOI:** 10.1093/jscr/rjaf465

**Published:** 2025-07-26

**Authors:** Eva Khalouf, Jad Shehadeh, Thourya Al Masri, Judy Alhakim, Mona Ayash, Zeina Shakouhy

**Affiliations:** Department of Pharmacy, Damascus University, Al Mazraa, Osama Bin Zaid Street, Damascus, Syrian Arab Republic; Department of Medicine, Damascus University, Al‑Mazzah Highway, Mazzeh District, Damascus, Syrian Arab Republic; Department of Medicine, Damascus University, Al‑Mazzah Highway, Mazzeh District, Damascus, Syrian Arab Republic; Department of Medicine, University of Kalamoon, Homs–Damascus International Highway, Deir Atiyah, Al-Nabk District, Rif Dimashq Governorate, Deir Attia, Syrian Arab Republic; Al Mouwasat University Hospital, General Surgery, Omar Bin Abdulaziz Street, Al Mazzeh District, Damascus, Syrian Arab Republic; Department of Medicine, Lattakia University, Aleppo Avenue, Latakia District, Lattakia, Syrian Arab Republic

**Keywords:** teratoma, pediatric, liver, case report

## Abstract

Teratomas are rare tumors composed of tissues from all three germ layers: ectoderm, mesoderm, and endoderm. While they are most commonly found in the gonads, they can also occur in extragonadal sites, including the liver—though this is extremely uncommon, accounting for < 1% of liver neoplasms. We report the case of a 14-year-old female who presented with right upper quadrant pain and nausea and was initially diagnosed with a hydatid cyst. However, surgical resection and histopathological analysis revealed a mature cystic teratoma of the liver. This case highlights the importance of considering teratomas in the differential diagnosis of liver masses, particularly when imaging findings are atypical. Surgical excision remains the treatment of choice, and histology plays a crucial role in confirming the diagnosis and guiding further management.

## Introduction

The name ‘teratoma’ comes from the Greek word ‘teratos,’ meaning ‘monster’ [1]. Teratomas are encapsulated tumors that are generally well-defined and can be readily distinguished from adjacent tissues. They consist of a mixture of tissues derived from the three germ layers, each of which can differentiate into various tissue types, replicating a wide range of bodily structures [2]. In oncology, this tridermal structure is unusual. Theoretically, every cell in the body has the potential for the complete range of cellular phenotypic expression, as each cell contains a full genome within its nucleus [3].

The ovaries and testes are the most common sites of teratomas, followed by the anterior mediastinum, retroperitoneum, sacrococcygeal area, and, less frequently, the skull. Liver and gastrointestinal tract teratomas are rarely reported, representing <1% of all teratomas. Additionally, only 11 cases of liver-originating teratomas have been reported in adult populations, underscoring their extreme rarity [1]. Less than 50 cases of primary hepatic teratomas have been identified in the literature [4].

Typical symptoms include abdominal pain, gastrointestinal and genitourinary disturbances, and lower extremity or vaginal edema due to lymphatic obstruction [2]. Tumor rupture can result in liquefied sebaceous contents leaking into the peritoneum [5].

Teratomas are often diagnosed preoperatively due to their characteristic imaging features. However, in some cases, the radiologic findings may be inconclusive. Histological examination provides a definitive diagnosis and evaluates for immature components, which is critical for treatment planning [4]. Surgery is typically the treatment of choice [5].

## Case report

A 14-year-old female presented with nausea and mild pain in the right upper quadrant and epigastric region, without fever, jaundice, or weight loss. The symptoms had persisted for 1 year. Her personal and family medical history was unremarkable, and the abdominal examination was normal. Serum biochemical tests, including aspartate aminotransferase, alanine aminotransferase, and bilirubin, were within normal limits. Anti-*Echinococcus granulosus* antibodies tested negative.

**Figure 1:**
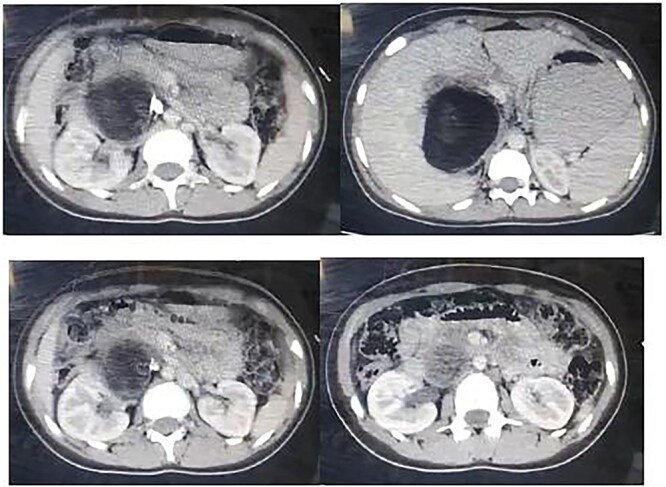
Non contrast CT showing a large hypogenous cyst of the caudate liver lobe lengthen to the hepatic hilum, dimensions of 7.7 × 9.5 cm with calcified composition of the wall.

Ultrasound revealed a large lesion in the caudate lobe of the liver with a hypoechoic, cystic structure and a thin wall, suggestive of an atypical type IV hydatid cyst. The lesion showed well-defined margins and scattered calcifications within the wall. A non-contrast computed tomography (CT) scan confirmed a large hypodense cyst in the caudate lobe extending to the hepatic hilum, measuring 7.7 × 9.5 cm, with calcified wall components (Fig. 1). No intrahepatic biliary dilation or lesions in other organs were observed.

**Figure 2:**
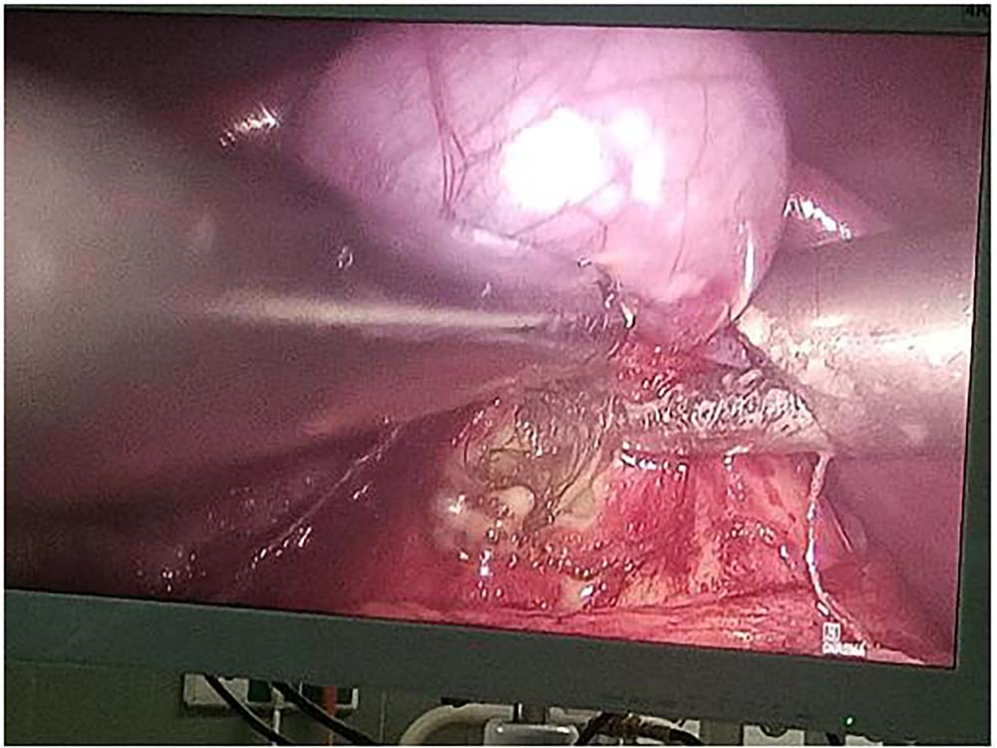
Intraoperative laparoscopic view demonstrating dissection of a hydatid cyst located in the caudate lobe of the liver. The cyst, consistent with an atypical type IV Echinococcus granulosus hydatid cyst, shows a well-defined, calcified wall. Atraumatic graspers are used to carefully mobilize the cyst from surrounding hepatic tissue near the hepatic hilum.

**Figure 3:**
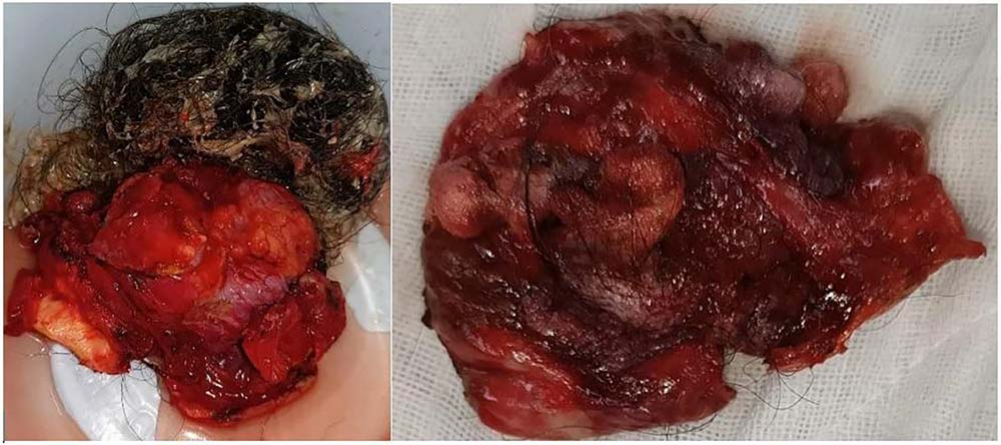
Resected specimen of the hepatic cystic mass, removed laparoscopically under vascular clamping. The excision was performed through healthy liver parenchyma, ensuring complete removal of the cyst wall. The gross appearance, featuring heterogeneous soft tissue components and hair-like structures, initially raised suspicion of a hepatic teratoma among the surgical team.

Following multidisciplinary consultation, the lesion was diagnosed as an *E. granulosus* hydatid cyst. Laparoscopic resection was planned (Fig. 2). Intraoperatively, surgeons suspected the cyst might represent a teratoma. Resection was performed under vascular clamping, with the cutting plane passing through healthy tissue, ensuring complete removal of the cyst wall (Fig. 3). The surgery was uneventful.

## Histopathology

**Figure 4:**
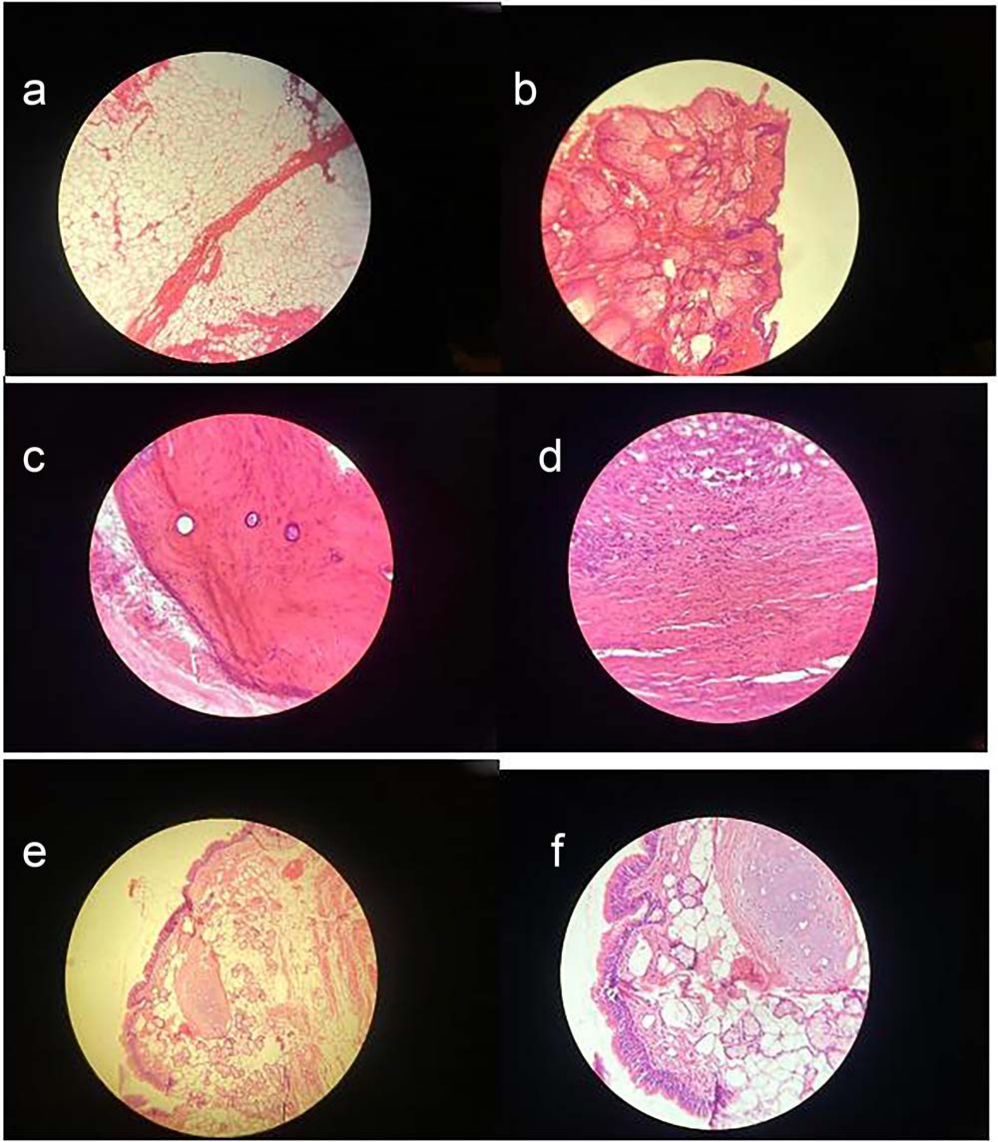
(a) Adipose tissue – This image shows large, empty-looking cells with thin borders, typical of adipocytes storing fat. (b) Squamous epithelium – Histological image showing stratified squamous epithelium (H&E stain, ×100), characterized by multiple layers of flattened cells. (c) Compact bone – This section reveals osteons with central (Haversian) canals surrounded by concentric lamellae. (d) Muscle – Histological image showing skeletal muscle tissue (H&E stain, ×100), with characteristic elongated, eosinophilic fibers and peripheral nuclei. (e) Skin adnexal structures – Histological image showing skin adnexal structures, including stratified squamous epithelium, sebaceous glands, and underlying connective and adipose tissue (H&E stain, ×100), consistent with ectodermal and mesodermal elements of a mature teratoma. (f) Cartilage tissue – H&E-stained section showing hyaline cartilage with chondrocytes in lacunae and a smooth matrix. Adjacent connective tissue and adipocytes indicate transition to surrounding structures.

**Figure 5:**
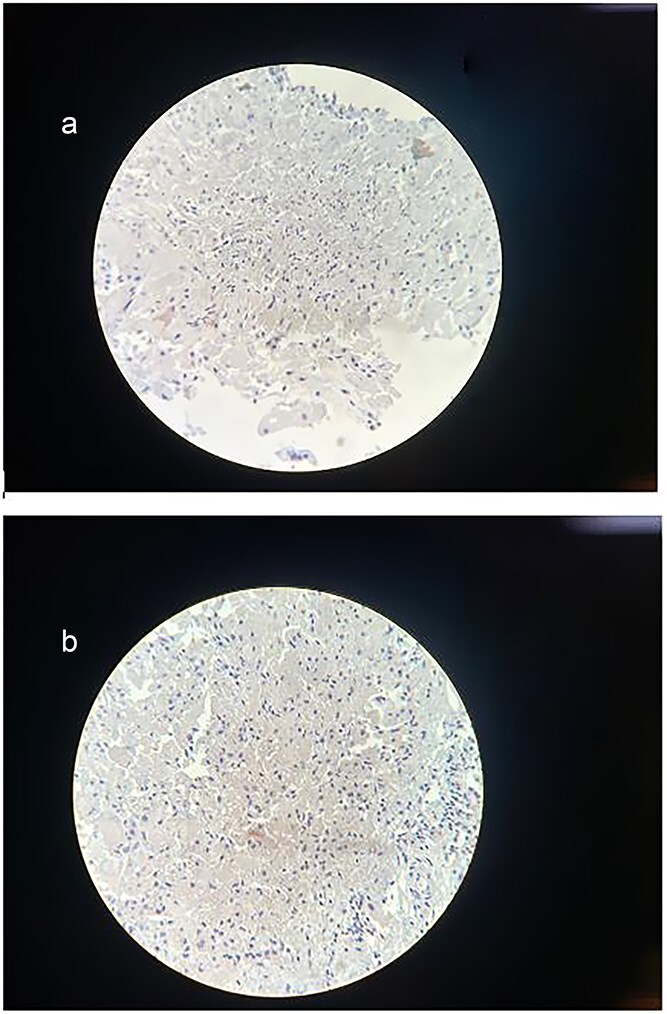
(a) Immunohistochemical staining for Synaptophysin. The sample shows a negative result for synaptophysin expression, excluding neuroendocrine differentiation within the lesion and further confirming the diagnosis of a mature cystic teratoma. (b) Immunohistochemical staining for Ki-67. The section demonstrates a low Ki-67 proliferation index, with minimal to no nuclear staining observed. This indicates low proliferative activity, supporting the diagnosis of a mature cystic teratoma.

Macroscopically, the resected lesion was described as a well-encapsulated cystic tumor measuring 90 × 85 mm, containing hair, fat, teeth, and cartilage (Fig. 4). The cyst wall was partially lined by keratinized stratified squamous epithelium (Fig. 5).

Immunohistochemical staining showed a negative result for synaptophysin, and the Ki-67 proliferation index was low, supporting the diagnosis of a mature cystic teratoma without neuroendocrine differentiation or significant proliferative activity.

The final diagnosis was mature cystic teratoma of the hepatic hilum.

## Discussion

Teratomas are neoplasms composed of tissues from the three germinal layers. The term ‘teratoma’ is derived from the Greek ‘teratos,’ meaning ‘monster.’ Germ cells, which later form the reproductive system, descend during fetal development. When they fail to migrate properly along the urogenital ridge, they may persist in extragonadal sites, leading to teratoma formation. Teratomas most commonly occur in the ovaries, followed by the testes, anterior mediastinum, retroperitoneum, sacrococcygeal area, and skull [6]. Teratomas of the liver are extremely rare, especially in adults, comprising <1% of liver tumors. They occur more frequently in females and are more common in the right hepatic lobe [7].

Immature liver teratomas typically present with right upper quadrant pain, fatigue, nausea, vomiting, jaundice, and weight loss [8]. In this case, the patient presented with nausea and right upper quadrant pain. There was no fever, jaundice, or weight loss, and liver function tests were normal.

Histologically, teratomas are classified as mature or immature, depending on the level of tissue differentiation [8]. Our patient was diagnosed with a mature teratoma with an immature focal component.

CT and magnetic resonance imaging are the primary imaging tools for identifying liver teratomas, revealing their size, composition, and relationship to adjacent structures. Some patients may exhibit elevated serum markers such as alpha-fetoprotein, lactate dehydrogenase, human chorionic gonadotropin, carcinoembryonic antigen, and liver enzymes. However, histological examination remains the gold standard for diagnosis [9].

In this case, initial imaging showed an oval cystic lesion in segment I of the liver, leading to a misdiagnosis of hydatid cyst.

Complete surgical excision remains the definitive treatment [10]. The cyst was removed laparoscopically, and upon aspiration, a large amount of pus was released. Hair and teeth were found upon opening the cyst wall. The procedure was converted to open laparotomy to ensure full resection.

### Postoperative course and follow-up

The patient recovered uneventfully. She was monitored in the General Surgery department and discharged 3 days postoperatively, after drain removal.

At the 2-week follow-up visit for suture removal and histopathology review, the patient was asymptomatic and had a normal abdominal exam. No additional imaging or lab tests were performed.

Unfortunately, the patient was lost to follow-up after this visit, precluding long-term monitoring. As a result, recurrence, delayed complications, or long-term outcomes could not be evaluated. This highlights the need for structured follow-up, especially in rare hepatic tumors. Nevertheless, this case adds valuable insight into the rare presentation and surgical management of hepatic teratomas in adolescents.

## Data Availability

Not applicable.
